# A critical brainstem relay for mediation of diffuse noxious inhibitory controls

**DOI:** 10.1093/brain/awad002

**Published:** 2023-01-10

**Authors:** Mateusz W Kucharczyk, Francesca Di Domenico, Kirsty Bannister

**Affiliations:** Central Modulation of Pain, Institute of Psychiatry, Psychology and Neuroscience, King’s College London, London SE1 1UL, UK; Laboratory of Neurophysiology, Department of Biochemical Toxicology, Chair of Toxicology, Faculty of Pharmacy, Jagiellonian University Medical College, Krakow 30-688, Poland; Central Modulation of Pain, Institute of Psychiatry, Psychology and Neuroscience, King’s College London, London SE1 1UL, UK; Central Modulation of Pain, Institute of Psychiatry, Psychology and Neuroscience, King’s College London, London SE1 1UL, UK

**Keywords:** noradrenaline, diffuse noxious inhibitory controls (DNIC), descending pain modulatory system (DPMS), wide dynamic range neurons, in vivo electrophysiology, optogenetics, pain inhibition

## Abstract

The CNS houses naturally occurring pathways that project from the brain to modulate spinal neuronal activity. The noradrenergic locus coeruleus (the A6 nucleus) originates such a descending control whose influence on pain modulation encompasses an interaction with a spinally projecting non-cerulean noradrenergic cell group. Hypothesizing the origin of an endogenous pain inhibitory pathway, our aim was to identify this cell group.

A5 and A7 noradrenergic nuclei also spinally project. We probed their activity using an array of optogenetic manipulation techniques during *in vivo* electrophysiological experimentation. Interestingly, noxious stimulus evoked spinal neuronal firing was decreased upon opto-activation of A5 neurons (two-way ANOVA with Tukey *post hoc*, *P* < 0.0001). Hypothesizing that this may reflect activity in the noradrenergic diffuse noxious inhibitory control circuit, itself activated upon application of a conditioning stimulus, we opto-inhibited A5 neurons with concurrent conditioning stimulus application. Surprisingly, no spinal neuronal inhibition was observed; activity in the diffuse noxious inhibitory control circuit was abolished (two-way ANOVA, *P* < 0.0001).

We propose that the A5 nucleus is a critical relay nucleus for mediation of diffuse noxious inhibitory controls. Given the plasticity of diffuse noxious inhibitory controls in disease, and its back and forward clinical translation, our data reveal a potential therapeutic target.

## Teaser

Diffuse noxious inhibitory control is a phenomenon whereby one noxious stimulus can inhibit the pain response to another. Kucharczyk *et al.* identify the A5 noradrenergic cell group as a critical brainstem relay in the descending pathway mediating this form of endogenous pain modulation.

## Introduction

The descending pain modulatory system (DPMS) comprises pathways that (i) emerge from distinct origin nuclei; and thus (ii) are subserved by discrete neuroanatomical frameworks. Housed in the brainstem, the A5, A6 and A7 nuclei all contain spinally-projecting noradrenergic neurons.^1,2^ The A6 nucleus, better known as the locus coeruleus (LC), originates an endogenous analgesic circuit historically linked to activation of spinal α_2_-adrenoceptors (ARs). The role of brainstem noradrenergic nuclei in pain modulatory processing is complex as evidence by the potentiated inhibitory effect of spinal α_2_-ARs antagonism on pain-related behaviours,^3^ opposing α_2_-AR-mediated facilitatory signalling in the brainstem,^4^ and the modular functional organization of the LC coupled with its role as a chronic pain generator.^5,6^

We recently demonstrated that LC-modulation of spinal wide dynamic range (WDR) neuronal nociceptive processing is linked to an interaction with a non-cerulean noradrenergic cell group.^7^ Proposing that this interaction may underlie the role of the LC as a chronic pain generator, our present study sought to identify this non-cerulean noradrenergic cell group. Since WDR neurons govern plasticity in the transmission centre and are thus placed front and centre stage of mechanisms that can initiate the development of persistent pain, we examined their electrophysiological properties upon spatial and genetic manipulation of A5, A6 and A7 nuclei. Our aim was to evidence a critical brainstem relay for a hitherto undefined descending inhibitory pathway.

## Materials and methods

### Animals

Male Sprague-Dawley rats were used. All procedures were approved by the Home Office and adhered to the Animals (Scientific Procedures) Act 1986, International Association for Study of Pain^8^ and ARRIVE ethical guidelines.^9^

All experiments contained a minimum of six rats per group, based on G-power predictions from previous experiments.^7,10^ Animals were randomly assigned to experimental groups. 65 rats were assigned as follows: SC-canine adenovirus (CAV)/catecholaminergic-specific synthetic promoter PRSx8 (PRS)-GtACR2-fRed injected: A5 = 6, A6 = 7, A7 = 6 rats; SC-CAV/PRS-ChR2-mCherry injected: A5 = 10 rats; intersectional adeno-associated viruse (AAV) approach: (Jaws) A5 = 10 rats, A6 = 7 rats, and A7 = 6 rats; additionally, 13 naïve rats were used for pharmacology. In total 91 deep dorsal horn (DDH) WDR neurons were recorded from 65 rats in 113 experimental approaches (listed on the spreadsheet in the Supplementary material).

### Descending noradrenergic neuron transduction

Two approaches were implemented to transduce spinally projecting catecholaminergic brainstem neurons: (i) CAV carrying channelrhodopsin-2 (ChR2) or *Guillardia theta* anion-conducting channelrhodopsin-2 (GtACR2)^11^ under the control of catecholamine-specific synthetic promoter (sPRS)^12^ (PVM) was injected unilaterally in the lumbar spinal cord globally transducing descending noradrenergic neurons *in situ*^3,13^; and (ii) AAV retrograde vectors (AAVrg^14^) carrying floxed red-shifted cruxhalorhodopsin, Jaws (Addgene), were microinjected spinally, and a minimum of 1 week later, a second AAV9 vector encoding Cre recombinase under tyrosine hydroxylase (TH) promoter (Addgene) was microinjected into the A5, A6 or A7 nucleus ipsilateral to the spinal injection (to restrict labelling to catecholaminergic neurons *in situ*).

### Spinal cord *in vivo* electrophysiology

*In vivo* electrophysiology was performed on animals weighing 240–300 g as previously described.^10^ Physiological homeostasis was monitored throughout. Extracellular single-unit activity of spinal WDR neurons in deep laminae IV/V was measured. Mechanical stimuli (8, 26 and 60 g von Frey filaments) and von Frey filaments with concurrent ipsilateral calibrated noxious ear pinch [to trigger diffuse noxious inhibitory controls (DNIC)^10^], were applied to the receptive field for 10 s per stimulus. DNIC are quantified as the inhibitory effect of noxious ear pinch on WDR neuronal firing (% of inhibition after ear pinch). Following baseline control data collection, 100 μg atipamezole (an α_2_-AR antagonist), or 20 μg prazosin hydrochloride (α_1_-AR antagonist) was administered topically on the lumbar spinal cord surface.

### Optogenetics

ChR2 was activated using a 450 nm laser (20 ms pulse at 5 Hz, 238 mW/mm^2^). Continuous irradiation (400 mW/mm^2^) was used to activate GtACR2. Continuous 637 nm laser (160 mW/mm^2^) was used to activate Jaws. Light power density was measured at the tip of the implantable 200 μm fibre cannula.^3^ Spinal WDR neurons were characterized by three stable baseline responses followed by three optically modulated responses. For combined optogenetics and spinal pharmacology, after collecting three stable baseline and three stable optoactivation responses, a drug was applied on the cord surface. On completion, animals were sacrificed by isoflurane overdose and transcardially perfused with saline followed by 4% paraformaldehyde.

### Immunohistochemistry

Cryosected tissue was incubated with primary antibodies against dopamine-β-hydroxylase (DBH: 1:500, Millipore), mCherry (1:500, Abcam), fRed (1:500, Evrogen), or eGFP (1:1000, Abcam) followed by appropriate fluorophore-conjugated secondary antibodies. Samples were imaged with an LSM 710 laser-scanning confocal microscope (Zeiss) and analysed with Fiji Win 64. For quantification, samples were imaged with Zeiss Imager Z1 microscope. Six to eight slices were imaged per animal. Cell counting was carried out on the Fiji Win 64 utilizing cell counter plugin. On average, 25 brainstem sections were imaged for quantification.

### Passive tissue clearing

A passive CLARITY tissue clearing technique (PACT) was implemented to allow imaging of thick (1000–2500 µm) spinal cord and brainstem fragments.^15^ Anti-fRed (1:500, Evrogen) primary antibody was used followed by Alexa Fluor 647 secondary antibody (1:500, Invitrogen). Samples were imaged with Zeiss LSM 780 confocal upright microscope. Scans typically spanned 400–700 μm. Images were analysed with Zen 2012 Blue Edition software followed by Fiji (ImageJ) equipped with appropriate plugins.

### Quantification and statistical analysis

Typically, three WDR neurons were characterized per preparation (*n*), and data were collected from at least six rats per group (*N*). Pharmacological investigation was performed on one neuron per animal. Statistical analysis was performed either on *n* for populational studies, or *N* for pharmacological studies. Uncorrected two-way repeated-measures (RM) ANOVA with the Tukey *post hoc* was used to assess von Frey and DNIC responses in the baseline conditions. For pharmacological experiments, Geisser-Greenhouse correction was used for RM-ANOVA. GraphPad Prism was used to analyse the data. *P* < 0.05 was considered significant.

### Data availability

Means of stimulus-evoked responses for each studied neuron are provided in the Supplementary material. Full length Spike2-format recordings as well as other data are available upon request.

## Results

### Spinal α_2_-adrenoceptors mediate DNIC

**Figure 1:**
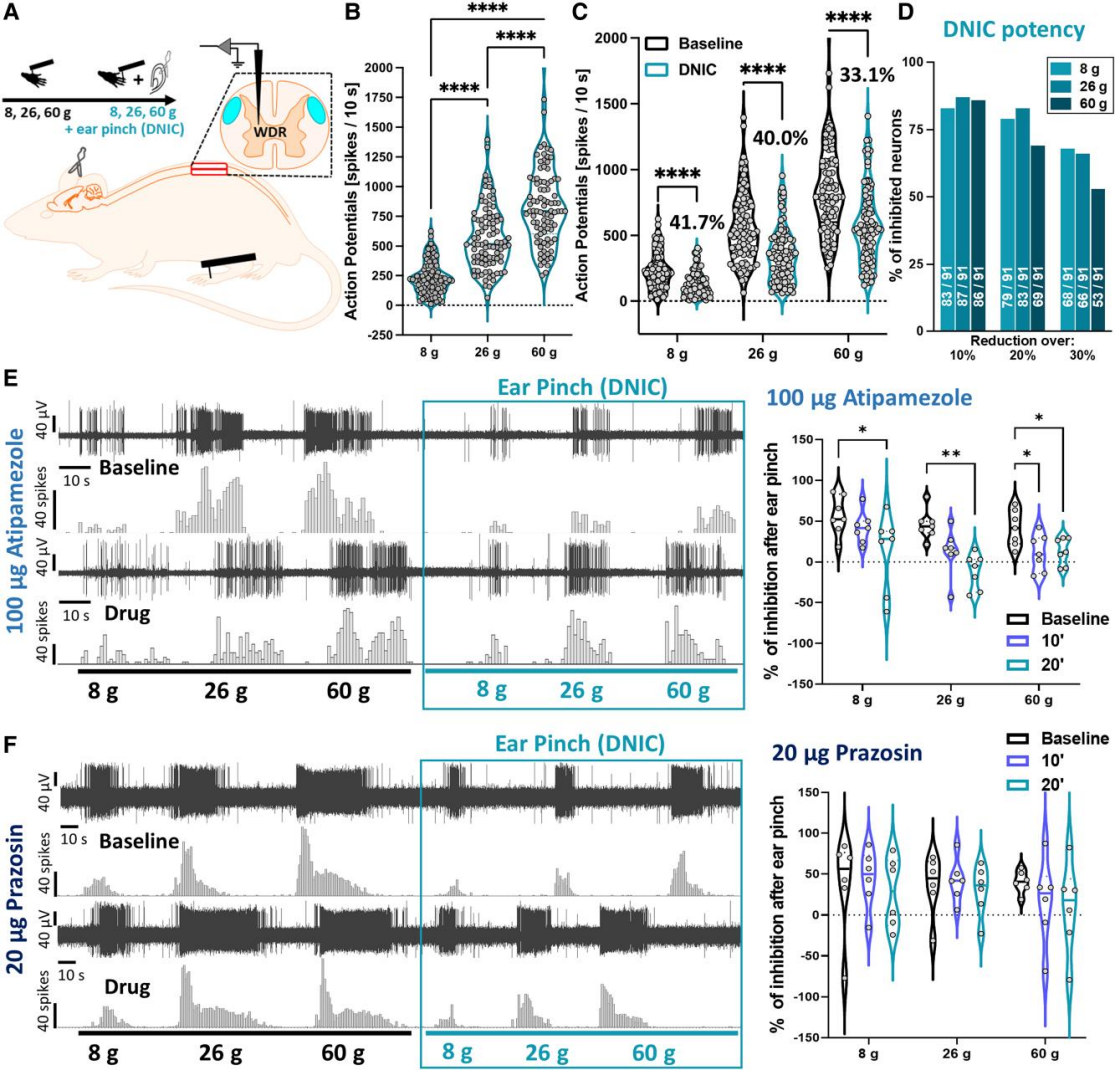
**Spinal α_2_-adrenoceptors mediate DNIC**. (**A**) Experimental setup. (**B**) DDH-WDR neurons code stimulus intensity (von Frey-evoked). (**C**) Application of noxious ear pinch (conditioning stimulus, CS) leads to inhibition of DDH-WDR firing. (**D**) Percentage of neurons inhibited by CS. Numbers on bars represent units with reduced activity according to a given threshold (out of 91 recorded). (**E**) Inhibition following CS application (baseline and following α_2_-adrenoceptor antagonism with spinal atipamezole) with example single unit DDH-WDR neuronal traces. (**F**) Inhibition following CS application (baseline and following α_1_-adrenoceptor antagonism with spinal prazosin) with example single unit DDH-WDR neuronal traces. Data represents mean ± SEM. Dots represent individual neuron studied (Baselines: *N* = 65 rats, *n* = 91 neurons). For pharmacology one cell was recorded per animal (atipamezole: *N*/*n* = 7, prazosin: *N*/*n* = 6). Two-way RM-ANOVA with Tukey *post hoc*: *P < 0.05, **P < 0.01, ****P < 0.0001. See Supplementary Fig. 1.

The functional expression of DNIC was recorded in healthy anaesthetized animals using terminal electrophysiological recordings of 91 polymodal and intensity coding [RM-ANOVA with Geisser-Greenhouse correction: (von Frey) *F*(1.39,24.40) = 363.8, *P* < 0.0001, Tukey *post hoc*] (Fig. 1A and B) lumbar deep dorsal horn WDR neurons (mean depth 852.7 ± 7.1 μm) (Supplementary Fig. 1A). WDR-evoked firing rates were significantly decreased upon simultaneous application of a conditioning stimulus (CS) [RM-ANOVA: (DNIC) *F*(1,90) = 505.2, *P* < 0.0001, Tukey *post hoc*] (Fig. 1C). Specifically, application of CS resulted in 41.7%, 40.0% and 33.1% inhibition of the evoked action potentials to 8, 26 and 60 g von Frey application, respectively (Fig. 1C), thus achieving a reduction greater than 20% for all forces tested (Fig. 1D). Spinal application of α_2_-AR antagonist atipamezole^16^ abolished DNIC expression [100 μg atipamezole: two-way RM-ANOVA: *F*(1.38,8.26) = 15.19, *P* < 0.01, Tukey *post hoc* test] (Fig. 1E). Spinal application of α_1_-AR antagonist prazosin^16^ failed to abolish DNIC [20 μg prazosin: two-way RM-ANOVA: *F*(1.03,5.12) = 0.57, *P* > 0.05] (Fig. 1F). Neither atipamezole [two-way RM-ANOVA: *F*(1.13,6.78) = 0.314, *P* > 0.05], nor prazosin [two-way RM-ANOVA: *F*(1.19,5.96) = 0.34, *P* > 0.05] impacted basal von Frey-evoked responses (Supplementary Fig. 1B and C).

### Inhibition of the dorsolateral funiculus abolishes diffuse noxious inhibitory controls

**Figure 2:**
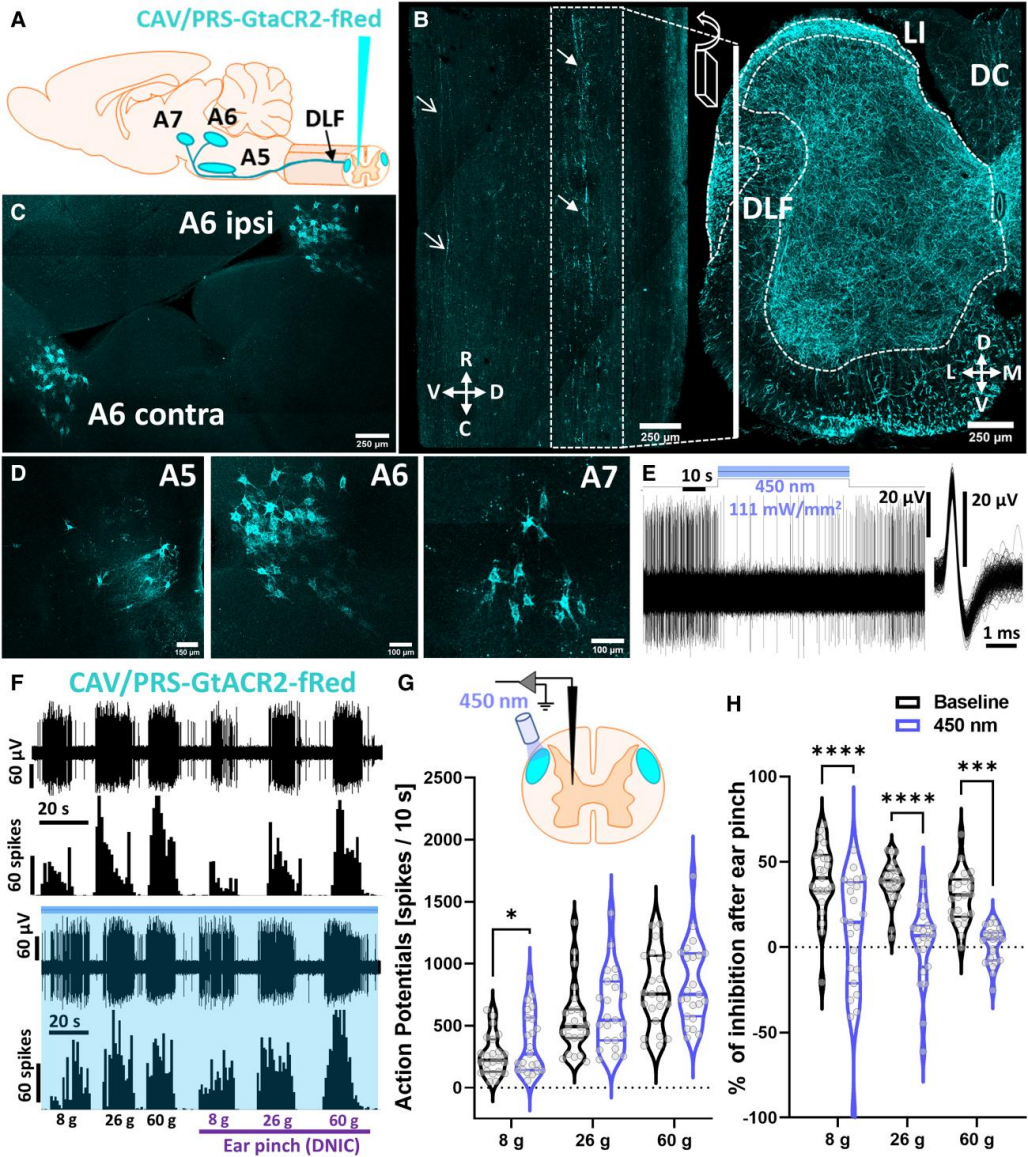
**Inhibition of the DLF abolishes DNIC**. (**A**) Experimental approach with CAV-PRS-GtACR2-fRed virus injected in the lumbar dorsal horn (DH) labelling discreet brainstem noradrenergic neuronal populations (A5, A6, A7). (**B**) 3D reconstruction of the light-transparent (PACT-cleared) 800-μm thick sagittal and coronal section of lumbar spinal cord evidencing labelled fibres travel via DLF. Closed arrows point at DLF, open arrows point at fibres in the ventrolateral funiculus. R = rostral, C = caudal, M = medial, L = lateral, V = ventral, D = dorsal, LI = lamina I, DC = dorsal column. (**C**) 3D reconstruction of the light-transparent 600-μm thick pontine coronal section showing bilateral labelling of the A6 coerulean neurons following unilateral virus injection in the lumbar DH. (**D**) As in **C**, zoomed on the A5, A6 and A7 spinally projecting noradrenergic neurons. (**E**) Representative single unit neuronal recording of the A6 GtACR2-expressing neuron inhibition following 450 nm continuous laser light illumination (400 mW/mm^2^). The inclusion shows overlay of 64 action potentials. (**F**) Example traces of von Frey-evoked firing of the DDH-WDR neurons before and after GtACR2-mediated inhibition (450 nm continuous laser light illumination, 400 mW/mm^2^, blue shaded) of the ipsilateral DLF. (**G**) Noxious (26 g, 60 g) von Frey-evoked firing of DDH-WDR neurons is not affected by the DLF optical inhibition. (**H**) DNIC, triggered by application of noxious ear pinch, are abolished after DLF GtACR2-mediated inhibition. Data represent mean ± SEM. Dots represent individual neuron studied (*N* = 18 rats, *n* = 23 neurons). Two-way RM-ANOVA with Tukey *post hoc*: *P < 0.05, ***P < 0.001, ****P < 0.0001. See Supplementary Figs 1 and 2 and Supplementary Video 1 (the video is available from figshare at https://doi.org/10.6084/m9.figshare.21786215).

The source of spinal noradrenaline is exclusively supraspinal. Noradrenergic fibres travel majorly via the dorsolateral funiculus (DLF).^13,17^ We retrogradely labelled descending projections to the lumbar spinal cord using spinally injected CAV with a construct expression restricted to catecholaminergic neurons by artificial PRS promoter (Fig. 2A–D and Supplementary Fig. 1D–G). The source(s) of lumbar noradrenaline were verified by the pathways’ reconstruction using optically transparent (PACT clearing^15^) tissue sections confirming the primarily DLF fibre route (Fig. 2B; some fibres were also detected in the ventrolateral funiculi), and efficiently labelling pontine A5-A7 noradrenergic somas (Fig. 2C and D and Supplementary Fig. 1D and E). Despite unilateral virus injection in the cord parenchyma, the labelling was bilateral with a bias towards ipsilateral dominance (Supplementary Fig. 1E–G).^13^ The labelled circuits were further confirmed as noradrenergic by immunostaining for DBH (Supplementary Fig. 1F). Double-labelled neurons were identified exclusively in the pontine A5, A6 and A7 nuclei (CAV: 24%, 12.7%, 16.6%—percentage of all DBH + neurons therein, respectively) (Supplementary Fig. 2G) corresponding with previous reports.^13,17–20^ Using microoptrode *in vivo* electrophysiological recordings of transduced A6 neurons we confirmed minimal light parameters for optogenetic manipulation (Fig. 2E).^7^

Since most fibres travelled via the ipsilateral DLF to later bifurcate (medullary decussation), we positioned a 200 µm optic fibre directly above the DLF 300–400 µm rostral to the recorded WDR neurons in rats expressing *G. theta* anion-conducting channelrhodopsin-2 (GtACR2),^11^ to inhibit descending noradrenergic controls with blue light.^21^ Given the short, transient effect of paradoxical activation upon GtACR2 axonal illumination as reported by others,^11,22^ we delivered continuous 450 nm laser illumination to the DLF (400 mW/mm^2^) at least 20 s prior and throughout our sensory testing (maximum 5 min). Interestingly, the DLF’s optoinhibition affected only innocuous (8 g), but not noxious (26 g and 60 g), von Frey-evoked basal firing of WDR neurons [two-way RM-ANOVA: *F*(1,22) = 4.46, *P* < 0.05, Tukey *post hoc* test: (8 g): *P* < 0.05, (26 g, 60 g): *P* > 0.05], suggesting the presence of a tonic noradrenergic inhibitory control restricted to innocuous mechanical stimuli (Fig. 2F and G). DLF inhibition resulted in an almost complete reversal of the DNIC effect [two-way RM-ANOVA: *F*(1,22) = 80.60, *P* < 0.0001, Tukey *post hoc* test: (8 g, 26 g): *P* < 0.0001, (60 g): *P* < 0.001] (Fig. 2F and H).

### Inhibition of spinally projecting A5 neurons abolishes DNIC

**Figure 3:**
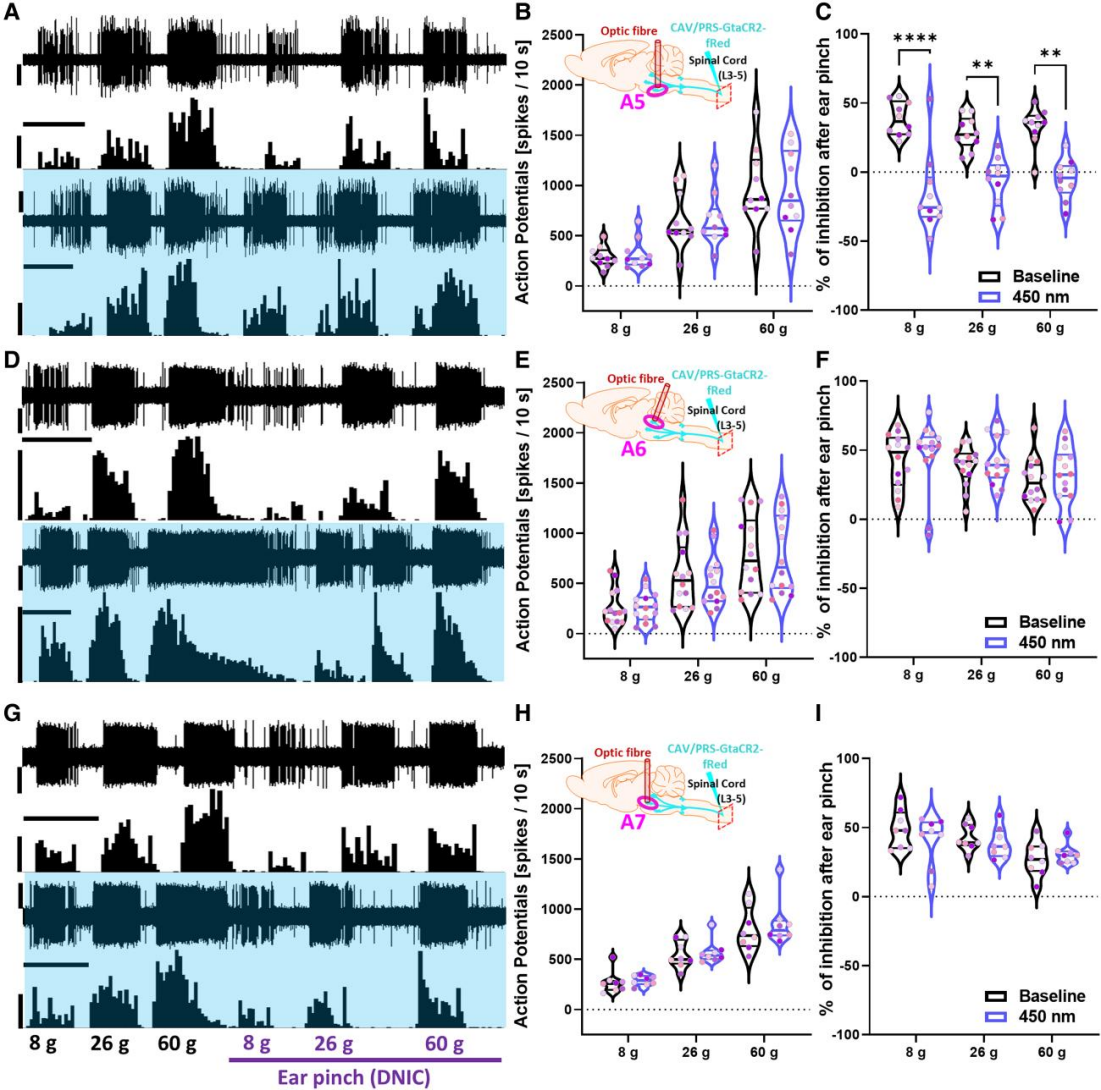
**Inhibition of spinally projecting A5 neurons abolishes DNIC**. (**A**, **D** and **G**) Example traces of DDH-WDR evoked neuronal firing before and after GtACR2-mediated inhibition (450 nm continuous laser light illumination, 400 mW/mm^2^) of labelled A5, A6, and A7 nuclei, respectively. (**B**, **E** and **H**) The basal evoked response of DDH-WDR neurons were not altered upon optical inhibition of A5, A6, or A7 nuclei. (**C**) DNIC were abolished after A5 GtACR2- but not A6 or A7 mediated inhibition, **F** and **I**, respectively. Data represents mean ± SEM. Dots represent individual neurons (A5: *N* = 6 rats, *n* = 10 neurons, A6: *N* = 7, *n* = 14, A7: *N* = 6, *n* = 8), and dots are colour coded to reflect neurons studied from the same animal. Two-way RM-ANOVA with Tukey *post hoc*: **P < 0.01, ****P < 0.0001. Scale bars in **A**, **D** and **G**: waveform trace = 60 μV; spike count = 60 spikes; time scale = 20 s. See Supplementary Figs 3 and 4.

Following robust labelling of the A5-A7 brainstem nuclei with the CAV vectors each nucleus was selectively illuminated in separate animals via a sterotaxically implanted optic fibre positioned 200 µm above the target nucleus (Supplementary Fig. 2A–C). Optic fibres used to target each nucleus were paint-coated except for the tip, to ensure minimum light off target effects around the fibre. Using this spatially and genetically restricted approach, we found that inhibition of no nuclei affected basal mechanically-evoked activity of spinal DDH-WDR neurons: [A5: two-way RM-ANOVA (450 nm) *F*(1,9) = 0.022, *P* > 0.05; A6: two-way RM-ANOVA (450 nm) *F*(1,13) = 0.203, *P* > 0.05; A7: two-way RM-ANOVA (450 nm) *F*(1,7) = 0.806, *P* > 0.05] (Fig. 3A, B, D, E, G and H). Interestingly, only inhibition of spinally projecting A5 noradrenergic neurons by direct illumination of their somas abolished DNIC expression [two-way RM-ANOVA (DNIC) *F*(1,9) = 107.8, *P* < 0.0001, with Tukey *post hoc*: (8 g): *P* < 0.0001, (26 g, 60 g): *P* < 0.01] (Fig. 3A and C). Neither inhibition of the A6 nor the A7 cell groups had any effect on DNIC expression [A6: two-way RM-ANOVA (DNIC) *F*(1,13) = 0.958, *P* > 0.05; A7: two-way RM-ANOVA (DNIC) *F*(1,7) = 0.806, *P* > 0.05] (Fig. 3D, F, G and I).

### A5 neurons project directly to spinal lamina V and their activation inhibits spinal neuronal responses

**Figure 4:**
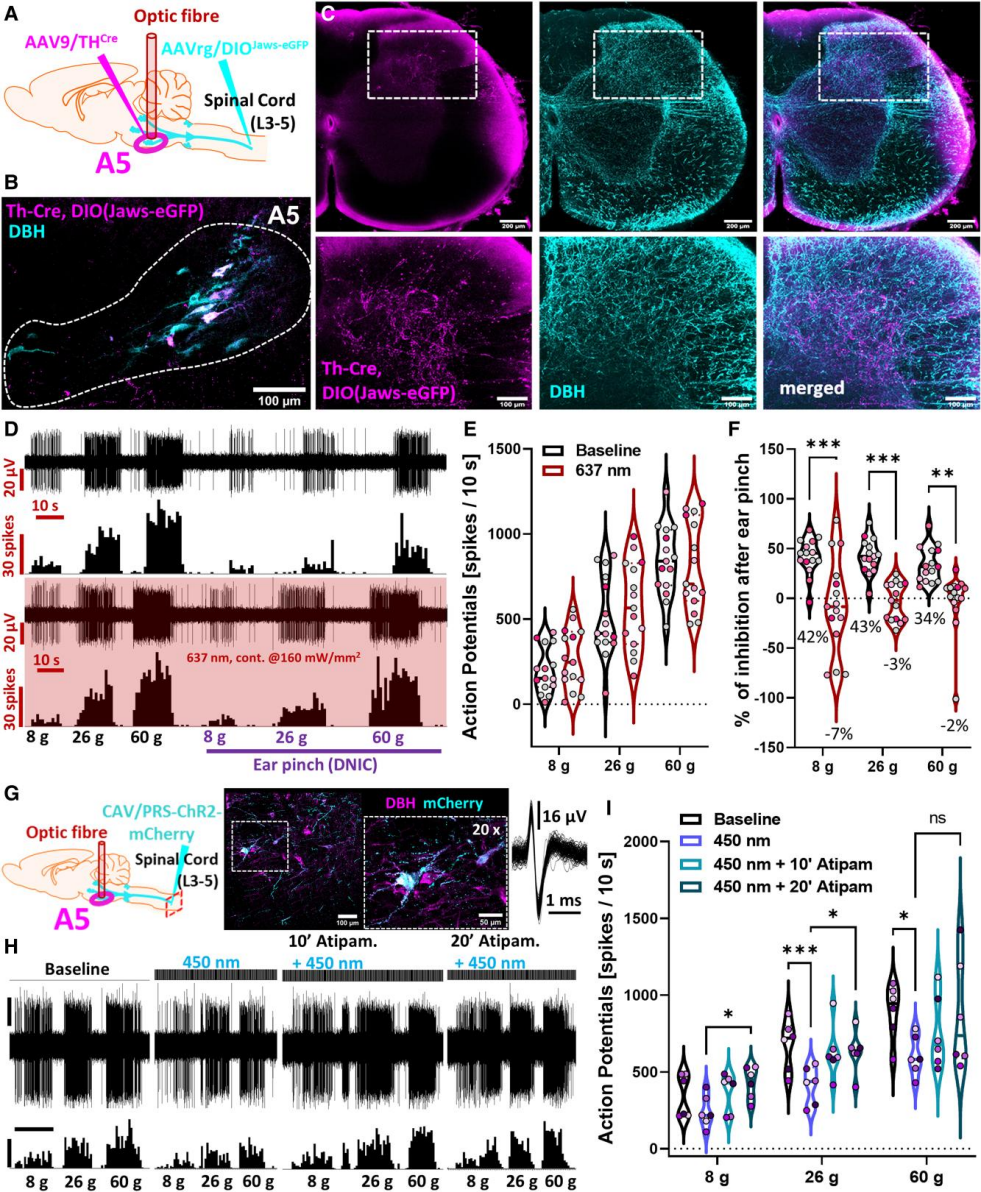
**A5 neurons project directly to spinal lamina V and their activation inhibits spinal neuronal responses**. (**A** and **B**) Experimental approach with immunohistochemical representation of the labelled nucleus. Two AAV intersectionally labelled discreet A5 brainstem noradrenergic (DBH) neuronal populations projecting to the lumbar spinal cord. (**C**) Light-transparent (PACT-cleared) 800-μm thick coronal section of lumbar spinal cord evidence accumulation of intersectionally labelled fibres in DDH laminae IV-V. (**D**) Example traces of the DDH-WDR neuron-evoked firing before and after Jaws-mediated inhibition (637 nm continuous laser light illumination, 160 mW/mm^2^) of the labelled spinally projecting A5 neurons. (**E**) DDH-WDR neuronal firing is not impacted by opto-inhibition of the A5 nucleus. (**F**) DNIC, triggered by application of noxious ear pinch (conditioning stimulus, CS), are abolished after A5 Jaws-mediated inhibition. (**G**) Experimental approach with immunohistochemical representation of the labelled A5 nucleus. CAV-PRS-ChR2-mCherry virus injected in the lumbar dorsal horn labels discreet brainstem A5 noradrenergic (DBH) neuronal populations. An inclusion shows an overlay of 64 action potentials of the neuron in B). (**H** and **I**) DDH-WDR neurons von Frey-evoked firing is inhibited following optoactivation (238 mW/mm^2^ 450 nm laser light 20 ms pulses at 5 Hz) of the ipsilateral A5 nucleus in a manner that is reversible following spinal α_2_-adrenoceptors antagonism by atipamezole. Dots represent individual neuron studied (in **I**): *N*/*n* = 6), and dots are colour coded to reflect neurons studied from the same animal. For pharmacology one cell was recorded per animal. Two-way RM-ANOVA with Tukey *post hoc*: *P < 0.05, **P < 0.01, ***P < 0.001. Scale bars in **H**: waveform trace = 30 μV; spike count = 60 spikes; time scale = 10 s. See Supplementary Fig. 5.

Next, we implemented an intersectional labelling approach for refined labelling. This approach efficiently labelled non-coerulean (A5/A7) spinal noradrenergic projections with low efficiency for the coerulean system (A6) (AAVrg: 39%, 1.1%, and 12.6%—percentage of all DBH + neurons in the A5, A6, and A7, respectively) (Fig. 4A and B and Supplementary Fig. 3A–F). Using PACT-cleared lumbar spinal cords, we evidenced that A5 fibres target DDH WDR-dwelling lamina IV-V (Fig. 4C), suggesting a direct A5 to DDH-WDR neuron projection.

Subsequently, using spatially and genetically restricted expression of red light (637 nm)-driven inward inhibitory chloride ion pump (Jaws), we verified that, as before, nucleus inhibition did not affect basal mechanically-evoked activity of spinal DDH-WDR neurons: [A5: two-way RM-ANOVA (637 nm) *F*(1,14) = 1.711, *P* > 0.05; A6: two-way RM-ANOVA (637 nm) *F*(1,10) = 0.353, *P* > 0.05; **A7**: two-way RM-ANOVA (637 nm) *F*(1,6) = 2.359, *P* > 0.05] (Fig. 4D and E and Supplementary Fig. 4A, B, D and E). Interestingly, only inhibition of spinally projecting TH + A5 neurons by direct illumination of their somas abolished DNIC expression [two-way RM-ANOVA (DNIC) *F*(1,14) = 39.09, *P* < 0.0001, with Tukey *post hoc*: (8 g, 26 g): *P* < 0.001, (60 g): *P* < 0.01] (Fig. 4D and F). Neither inhibition of the A6, nor the A7 cell groups had any effect on DNIC expression [A6: two-way RM-ANOVA (DNIC) *F*(1,10) = 0.295, *P* > 0.05; A7: two-way RM-ANOVA (DNIC) *F*(1,6) = 0.450, *P* > 0.05] (Supplementary Fig. 4A, C, D and F). Next, we adopted a previously optimized approach for CAV-mediated delivery of ChR2 to spinally projecting neurons from all three nuclei.^3,7,13^ After confirming a similar labelling pattern as for the GtACR2 constructs (Fig. 4G and Supplementary Fig. 5A and B), we optoactivated spinally projecting ChR2-expressing A5 neurons, with pulsed 450 nm laser light (5 Hz, 20 ms square-wave pulses at 238 mW/mm^2^). A5 optoactivation [two-way RM-ANOVA (450 nm) *F*(1,14) = 7.659, *P* < 0.05, Tukey *post hoc* test: (8 g): *P* > 0.05, (26 g): *P* < 0.001, (60 g): *P* < 0.0001] potently inhibited mechanically evoked DDH-WDR neuron firing in the absence of CS (Supplementary Fig. 5C and D), while optoactivation of the A5 nucleus had no effect on DNIC expression [A5: two-way RM-ANOVA (450 nm) *F*(1,11) = 1.02, *P* > 0.05]. A5-mediated DDH-WDR neuronal inhibition was reversed by spinal application of 100 μg atipamezole [two-way RM-ANOVA (drug) *F*(1.35,6.73) = 5.36, *P* < 0.05, Tukey *post hoc* test: (8 g, 26 g): *P* < 0.05, (60 g): *P* > 0.05], confirming an α_2_-AR-mediated mechanism of DNIC expression (Fig. 4H and I and Supplementary Fig. 5C and E).

## Discussion

Herein we investigated the impact of spatially and genetically restricted optical manipulation of descending projections from noradrenergic A5, A6 and A7 brainstem nuclei on spinal WDR neuronal activity. While inhibition of any singular nucleus did not inhibit basal WDR neuronal activity, activation of an excitatory opsin in the pontine A5 nucleus reduced the firing rate of spinal WDR neurons in a manner that was reversed by antagonism of the spinal α_2_-ARs. This led us to consider the mechanisms by which the body inhibits pain in an endogenous manner. DNIC, a naturally occurring pain-inhibiting pathway, is subserved by noradrenergic transmission via spinal α_2_-ARs.^7,10,23^ This pathway is not tonically active but rather is evoked by application of a CS concurrent to stimulation of WDR neuronal peripheral receptive fields. The origin of DNIC was initially postulated, following a series of lesioning experiments,^24,25^ to be the medullary reticular dorsal nucleus (MdD).^26^ However, a recent genetic, anatomically and functionally precise investigation revealed that activation of the MdD *Tac1^+^* neurons facilitates thermal pain reflexes.^27^ Further, the MdD is non-catecholaminergic. Interestingly, we found that upon activation of an inhibitory opsin on A5 neurons, WDR firing rates were no longer inhibited in response to application of a CS. Cumulatively, our data lead us to propose that the spinal cord projection site of a pathway that governs naturally occurring analgesia is the pontine A5 noradrenergic cell group, the origin of DNIC (Supplementary Fig. 5F).

Interestingly, optoinhibition of the DLF suggested the presence of a tonic noradrenergic inhibitory control restricted to innocuous mechanical stimuli. This result requires further investigation; given that inhibition of no individual nucleus replicated this effect, one interpretation is that there is an underlying interplay between the A-nucleus to spinal cord pathways. This could represent a homeostatic mechanism. For example, activation of the ipsilateral A6 is proalgesic via an interaction with superficial dorsal horn astrocytes expressing α_1_-ARs.^28^

Reciprocity between DPMS circuits that govern DNIC’s expression (and other modulatory controls) is highly likely and, in some cases, already evidenced.^7^ The fact that the DNIC pathway specifically inhibits the activity of WDR neurons, a cell group which (i) are so named because of their ability to respond differentially over a range of stimulus intensities; and (ii) famously underpin the gate control theory of pain, highlights that defining the functionality of pathways that directly modulate WDR activity is key for better understanding of the pain circuitry; delineation in health is necessary before dysfunction in disease may be pinpointed. Insight regarding brainstem and spinal α_2_-AR-mediated mechanisms, specifically linking DNIC attenuation to impairment of descending noradrenergic modulation from the LC in a rodent model of joint inflammatory pain,^29^ highlights the need to investigate governance of effects subsequent to A-nucleus activation in health and disease. Since screening for dysfunction in controls such as DNIC is clinically possible, tailored patient approaches could be on the horizon. Further preclinical investigation of the A5 nucleus is warranted.

## Supplementary Material

awad002_Supplementary_Data
