# Supplementary material for: A critical brainstem relay for mediation of diffuse noxious inhibitory controls
Source: Brain. 2023 Jan 10;146(6):2259–67. doi: 10.1093/brain/awad002 (PMC10232242; doi:10.1093/brain/awad002)
Supplement: awad002_Supplementary_Data [file awad002_supplementary_data.pdf]

KING'S College LONDON

University of London

Wolfson CARD

Central Modulation of Pain
The Wolfson Wing
Hodgkin Building
Guys Campus
London, SE1 1UL

SUPPLEMENTARY MATERIAL

A CRITICAL BRAINSTEM RELAY FOR MEDIATION OF DIFFUSE NOXIOUS INHIBITORY CONTROLS

MATEUSZ W. KUCHARCZYK, FRANCESCA DI DOMENICO, KIRSTY BANNISTER

Supplementary Figures and Tables

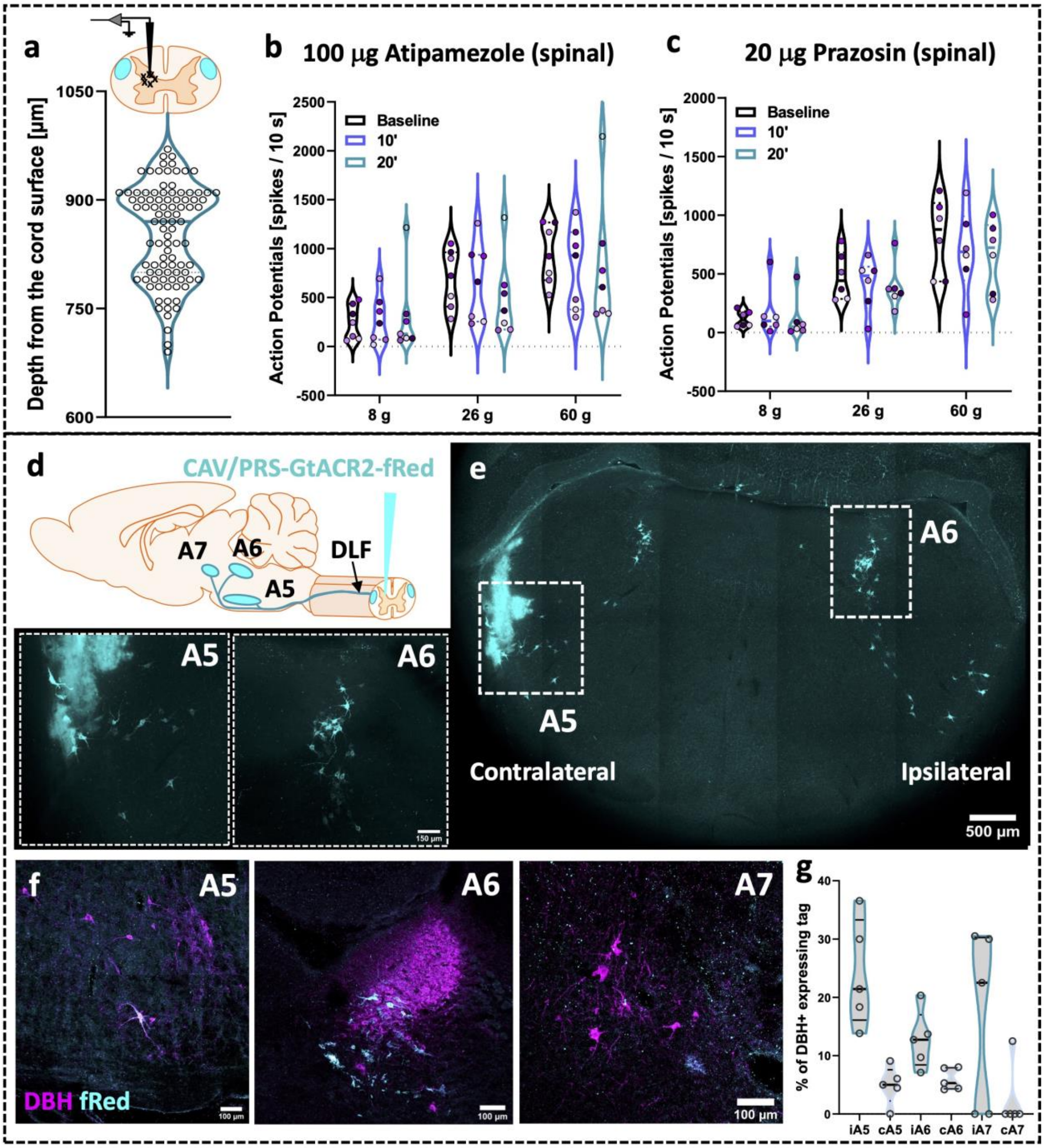

Figure S1. Spinal α_2-adrenoceptors mediate DNIC. a) Lumbar spinal cord electrode depths of all the recorded deep dorsal horn wide dynamic range (DDH-WDR) neurons. **b)** Quantification of von Frey-evoked action potentials before and after α_2-adrenoceptors block with spinal atipamezole, **c)** Quantification of von Frey-evoked action potentials before and after α_1-adrenoceptors block with spinal prazosin. **d)** Experimental approach with CAV-PRS-GtACR2-fRed virus injected in the lumbar dorsal horn (DH) labels discreet brainstem noradrenergic neuronal populations (A5, A6, A7). **e)** 3D reconstruction of the light-

transparent (PACT-cleared) 600 μm thick pontine coronal section showing bilateral labelling of the A5 and A6 neurons following unilateral virus injection in the lumbar dorsal horn. **f)** Immunohistochemical representation of the noradrenergic (dopamine-β-hydroxylase marked, DBH) labelled A5, A6 and A7 nuclei after CAV-PRS-GtACR2-fRed virus injected in the lumbar dorsal horn. **g)** Quantification of the CAV labelling efficiency shown as a percentage of all DBH+ neurons in a given ipsilateral to the virus injection nucleus (i-ipsi, c-contra). Data represents mean±SEM. For electrophysiology, dots represent individual neuron studied (all recorded neuron depths: N=69 rats, n=95 neurons). For pharmacology one cell was recorded per animal (atipamezole: N/n= 7, prazosin: N/n= 6), and dots are colour coded to reflect neurons studied from the same animal. Two-way RM-ANOVA. For histochemical quantification dots represent individual animal data as a mean from 6-8 brain slices per rat.

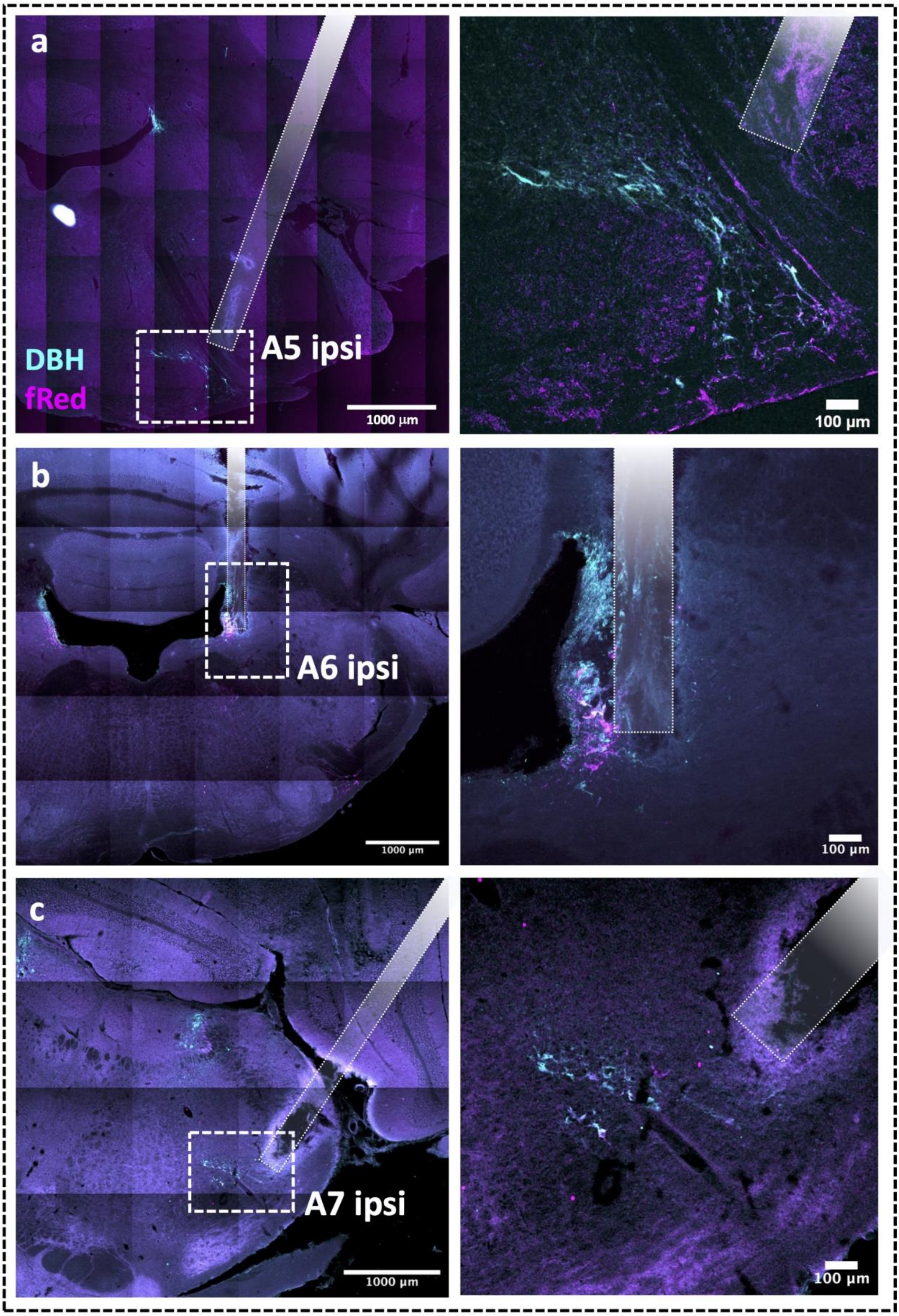

Figure S2. Example optic fibre tracks. Example tracks targeting brainstem **a)** A5, **b)** A6,

and **c)** A7 nuclei, ipsilateral to the recorded spinal neuron. DBH, dopamine-β-hydroxylase marked; fRed, fluorescent tag introduced by CAV/PRS-GtACR2-fRed injected in the ipsilateral lumbar spinal cord.

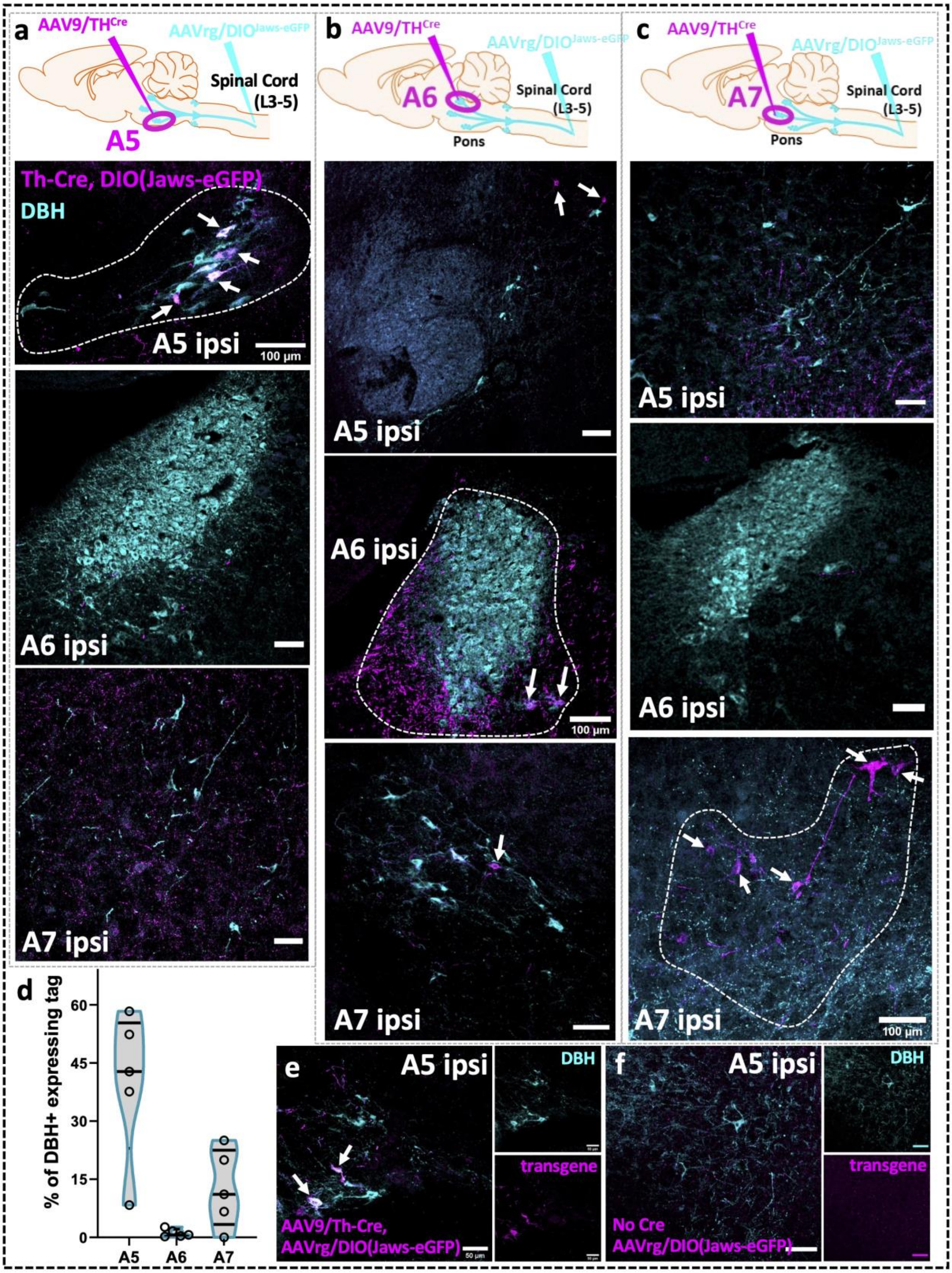

Figure S3. Intersectional viral strategy for labelling of discreet spinally projecting noradrenergic brainstem nuclei. Two different adeno-associated viruses (AAV) were used to intersectionally label discreet brainstem noradrenergic (dopamine-β-hydroxylase marked, DBH) neuronal populations projecting to the lumbar spinal cord (A5, A6, A7). In columns, experimental approach followed by immunohistochemical representation of the labelled A5, A6 and A7 nucleus for the AAV9/TH-Cre virus targeting **a)** A5 **b)** A6, and **c)** A7 nucleus. White arrows point at double labelled neurons. Note: A6 injection results in few neurons labelled also in the A5 and A7 nucleus. **d)** Percentage of double labelled DBH neurons expressing viral tag (eGFP) in A5-A7 nuclei after ipsilateral injection of AAV viruses. Note the low labelling efficiency for A6 coerulean neurons (<2%). Example of the A5 labelling **e)** with, and **f)** without (negative control) brainstem AAV9/TH-Cre virus injection in the A5 nucleus (analogous experiment to a); spinal virus was injected in both instances).

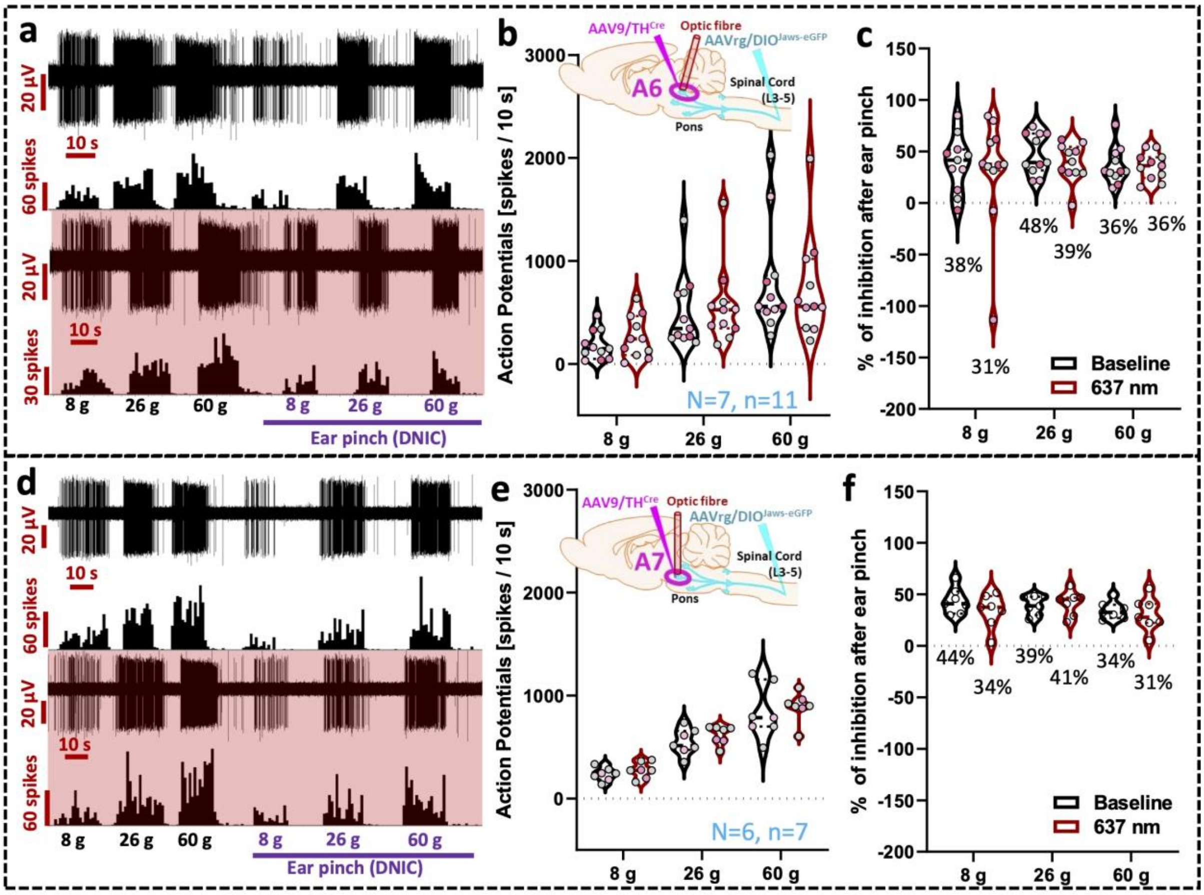

Figure S4. Jaws inhibition of A6 and A7 spinally projecting noradrenergic brainstem nuclei does not abolish DNIC. a, d) Example traces of the deep dorsal horn wide dynamic range (DDH-WDR) neurons von Frey-evoked firing before and after Jaws-mediated inhibition (637 nm continuous laser light illumination, 160 mW/mm^2) of the labelled spinally

projecting A6 and A7 neurons, respectively. **b, e)** DDH-WDR neurons von Frey-evoked firing is not affected by optical inhibition of the A6 or A7 nucleus, respectively. **c, f)** Diffuse noxious inhibitory controls (DNIC), triggered by application of noxious ear pinch (conditioning stimulus, CS), are not affected after A6 or A7 Jaws-mediated inhibition. respectively. Data represents mean±SEM. Dots represent individual neuron studied (A6: N=7 rats, n=11 neurons, A7: N=6 rats, n=7 neurons), and dots are colour coded to reflect neurons studied from the same animal. For histochemical quantification dots represent individual animal data as a mean from 6-8 brain slices per rat. Two-way RM-ANOVA.

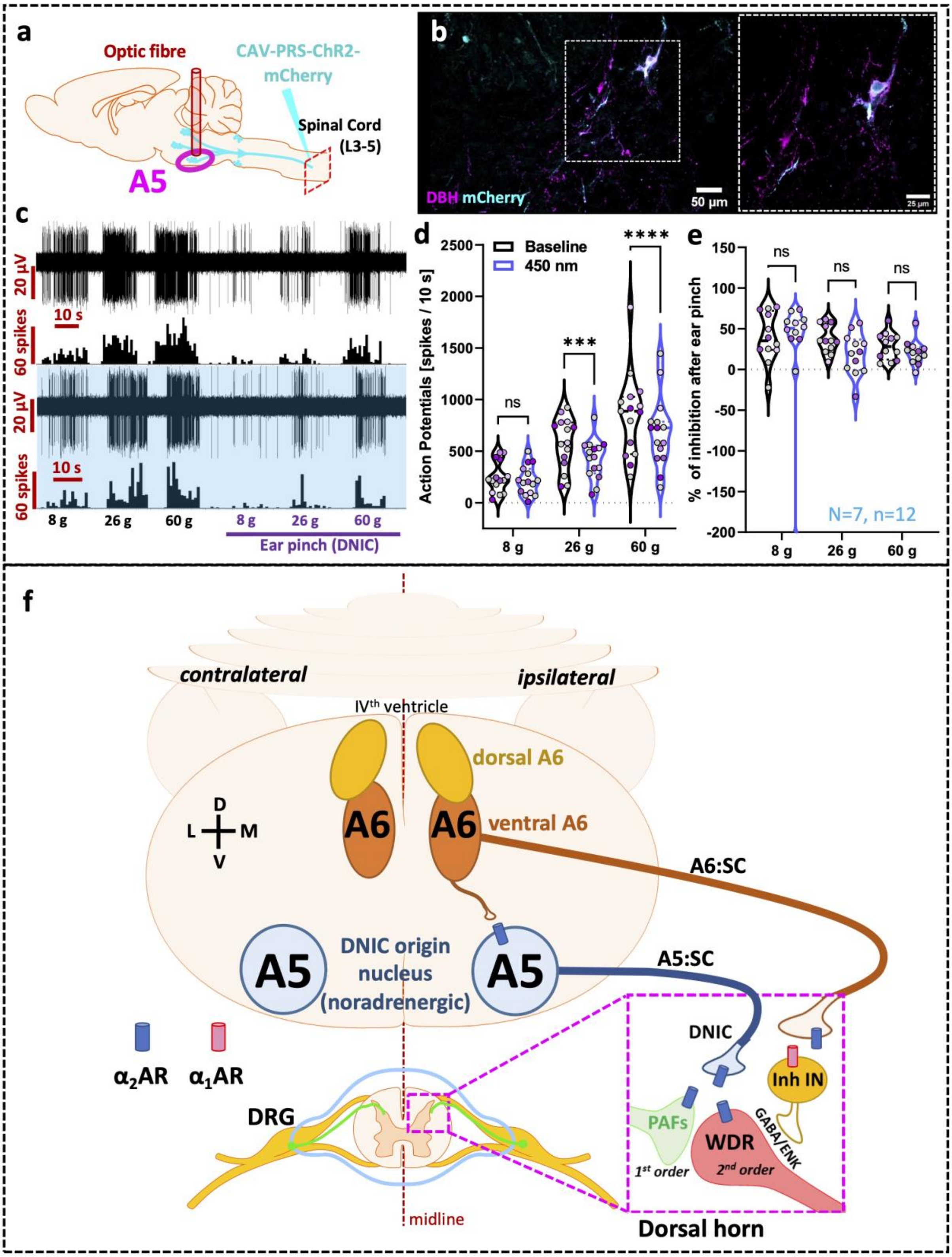

Figure S3. Optogenetic activation of spinally projecting A5 neurons inhibits spinal neuron activity. a, b) Experimental approach with immunohistochemical representation of the labelled A5 nucleus. CAV-PRS-ChR2-mCherry virus injected in the lumbar dorsal horn labels discreet A5 brainstem noradrenergic (dopamine-β-hydroxylase marked, DBH)

neuronal population **c)** Deep dorsal horn wide dynamic range (DDH-WDR) neurons von Frey-evoked firing is inhibited following optoactivation (238 mW/mm^2 450 nm laser light 20 ms pulses at 5 Hz) of ipsilateral spinally projecting A5 neurons. **d)** A5 optoactivation does not abolish diffuse noxious inhibitory controls (DNIC), triggered by application of noxious ear pinch, expression. Data represents mean±SEM. Dots represent individual neuron studied (N=7 rats, n=12 cells), and dots are colour coded to reflect neurons studied from the same animal. Two-way RM-ANOVA with Tukey *post-hoc*: ***P<0.001, ****P<0.0001. **f)** Schematic representation of the proposed circuits. Two parallel ipsilateral descending noradrenergic projections originate in the A5 and A6 brainstem nuclei, respectively, to modulate spinal nociceptive processing. While the A6:SC circuit is inhibitory, likely via indirect spinal inhibitory interneuron α_1-adrenoceptor (α_{1}-AR) mediated mechanism, the A5:SC circuit is likely inhibitory via direct via α_2-adrenoceptor (α_{2}-AR) activation. We propose that the A5:SC circuit is a critical relay conferring DNIC. Note that the A6 and A5 are suggested to crosstalk through brainstem relays.

Supplementary Materials and Methods

Animals

Male Sprague-Dawley rats (Envigo, UK) were used for experiments. Animals were group housed on a 12:12 h light–dark cycle. Food and water were available *ad libitum*. Animal house conditions were strictly controlled, maintaining levels of humidity (40–50%) and temperature (22 ± 2°C). All procedures described were approved by the Home Office and adhered to the Animals (Scientific Procedures) Act 1986. Every effort was made to reduce animal suffering and the number of animals used was in accordance with International Association for Study of Pain (IASP)[1] and ARRIVE ethical guidelines[2]. Aseptic surgical techniques were used throughout surgical interventions.

In this study we used 65 rats, assigned to the groups as follows: SC-CAV/PRS-GtACR2-fRed injected: A5=6, A6=7, A7=6 rats; SC-CAV/PRS-ChR2-mCherry injected: A5=10 rats; intersectional AAV approach: (Jaws) A5=10 rats, A6=7 rats, and A7=6 rats; additionally, 13 naïve rats were used for pharmacology. In total 91 DDH WDR neurons were recorded from 65 rats in 113 experimental approaches as listed in the supplementary data spreadsheet.

Virus injections

Spinal cord injections

Given animals exceeding 300 g cannot be used for our *in vivo* electrophysiological approach due to anatomical and rig electrophysiology frame constraints, we began our experiments with 60-70 g rats to allow for the animal growth, which after minimum of 2 weeks virus incubation would not exceed 260-290 g. Thus, rats weighing 60-70 g were anaesthetised using isoflurane (3–5% for induction and 1.5–2% for maintenance in 1 l/min oxygen flow, Piramal, UK), maintained at 37°C using a homeothermic blanket (Harvard Apparatus, US), and injected with 50 µl of Meloxicam (subcutaneous, 2 mg/kg, Metacam®, Boehringer Ingelheim, Berkshire, UK) for peri-operative analgesia. Animals were fixed in a stereotaxic apparatus (Kopf Instruments, UK), lumbar region was clamped, and T12-L1 intervertebral space was exposed by bending the region rostrally providing easy access to the underlaying dura and L3-4 spinal cord without the need for extensive laminectomy. For intersectional approach (paired with the brainstem injection, see next section): bilaterally, two paired injections (200 µm lateral from the midline and 750 µm apart rostro-caudally; first pair at 850

and second pair at 450 μm below the L3-L4 cord surface) of AAVrg viruses with improved retrograde axonal transport[3] were made to transduce descending spinal projections. Following AAV was used: AAVrg-CAG-FLEX-rc[Jaws-KGC-GFP-ER2] (titer >7x10^{12} vg/ml, viral prep #84445-AAVrg, Addgene, US,[4]). For global transduction of descending noradrenergic projections (no brain injection required, see below), three unilateral lumbar dorsal horn injections of canine adenovirus (CAV) were performed (200 μm lateral from the midline and 750 μm apart rostro-caudally; first at 850, second at 650, and third at 450 μm below the L3-L4 cord surface). For optogenetic activation CAV encoding for channelrhodopsin 2 under the control of catecholamine-specific synthetic promoter (PRS) was used (CAV/PRS-hChR2(H134R)-mCherry, titer >3x10^{10} TU/ml, PVM, Montpellier, a gift from Professor Anthony Pickering, University of Bristol[5,6]), while for inhibition CAV encoding for *Guillardia theta* anion-conducting channelrhodopsin (stGtACR2)[7,8] under PRS promoter was used (CAV/PRS-stGtACR2-FRed, titer >8x10^{10} TU/ml, PVM, Montpellier). Injections were made with a glass pulled micropipette (10-20 μm tip) coupled to electronically controlled nanoinjector (Nanoliter 2010, WPI, FL, US) facilitating precise delivery with minimal damage. Each injection was of 400 nl with 2 nl/s delivery rate and minimal 3-5 minutes wait between slow pipette retraction. The micropipettes were filled with inert mineral oil (extrusion medium), and the virus-oil interface was monitored to ensure injection. The wound was irrigated with saline and the incision was closed with wound clips and postsurgical glue (Vetabond, 3M, UK).

Brainstem injections

Brainstem injections were made on rats weighting 190-210 g (around 1-2 weeks after spinal cord surgery). Animals were anesthetized with i.p. ketamine (5 mg/100 g, Vetalar; Pharmacia) and medetomidine (30 μg/100 g, Dormitor; Pfizer) until loss of paw withdrawal reflex and perioperative analgesia was achieved by the s.c. injections of meloxicam (2 mg/kg). The rat was placed in a stereotaxic frame, with core temperature maintained using a homeothermic blanket. Using a 0.7 mm dental drill a hole was made in the skull directly above the targeted structure. The following coordinates were used: **A6 (locus coeruleus)**: 10° rostral angulation (to avoid puncturing the sinus), from lambda: RC: -2.1 mm, ML: +1.3 mm, and three injections at -6.2, -6.0, -5.8 mm deep from the cerebellar surface (2 mm deeper from lambda); **A5:** no angulation, from lambda: RC: -0.8 mm, ML: +2.4 mm, and three injections at -9.8, -9.6, -9.4 mm deep from lambda, **A7:** no angulation, from lambda: RC:

+0.1 mm, ML: +2.8 mm, and three injections at -7.8, -7.6, -7.4 mm deep from lambda. Three injections of AAV9/rTH-PI-Cre-SV40 (titer >7x10^{12} vg/ml, viral prep #107788-AAV9, Addgene, US, a gift from James M. Wilson) were made analogously to spinal injections. Each injection was of 300 nl, every 200 μm starting from deepest point chosen (DV) with 2 nl/s delivery rate and minimal 2-3 minutes between slow pipette retraction. 5 minutes after final injection the micropipette was retracted over the course of 4-5 minutes. The wound was irrigated with saline and closed with Vicryl 4-0 absorbable sutures and wound glue (VetBond 3M, UK). Anaesthesia was reversed with s.c. injection of atipamezole (Antisedan, 0.1 mg/100 g, i.p.; Pfizer). The animals were placed in a thermoregulated recovery box until fully awake. Two to three weeks were allowed for the transgene(s) expression.

Spinal Cord In Vivo Electrophysiology

In vivo electrophysiology was performed on animals weighing 240–300 g exactly as described previously[9]. Briefly, tracheotomised rats were maintained with 1.5% of isoflurane in a gaseous mix of N_2O (66%) and O_2 (33%) with core body temperature and electrocardiogram (ECG) monitored and maintained at physiological levels. Craniotomy was performed to gain stereotaxic access to the ipsilateral A5, A6 or A7 nuclei for optic fibre insertion as described in following sections. A laminectomy was performed to expose the L3–L5 segments of the spinal cord, and using a parylene-coated, tungsten electrode (125 μm diameter, 2 MΩ impedance, A-M Systems, WA, USA), wide dynamic range neurons in deep laminae IV/V (~650–900 μm from the dorsal surface of the cord) receiving intensity-coding afferent A-fibre and C-fibre input from the hind paw were sought by periodic light tapping of the glabrous surface of the hind paw. Single unit extracellular potentials were amplified (100-150k times) and filtered (bandwidth 1000 Hz to 5 KHz) using Neurolog system and digitalised signal (Power 1401 625kHz, CED) was fed to a PC running Spike2 v8.02 software (Cambridge Electronic Design, Cambridge, UK)) for data acquisition, analysis and storage. Stimulus histograms were built in real time along the waveform recordings. Simultaneous ECG monitoring and transistor–transistor logic (TTL) triggers (i.e. for the lasers, see below) were additionally coupled to Spike2 recording traces via CED-1401 analogue inputs.

Stimulation paradigm in all electrophysiological recordings

Natural mechanical stimuli consisting of von Frey filaments (8 g, 26 g and 60 g) alone followed by von Frey filaments with concurrent ipsilateral noxious ear pinch (15.75 × 2.3 mm Bulldog Serrefine ear clip; InterFocus, Linton, United Kingdom), were applied in this

order to the receptive field for 10 s per stimulus. The noxious ear pinch was used as a conditioning stimulus (CS) to trigger diffuse noxious inhibitory control (DNIC,[9–11]), and was quantified as an inhibitory effect on neuronal firing during ear pinch to its immediate respective von Frey filament applied without the conditioning stimulus (% of inhibition after ear pinch). A minimum 30 s non-stimulation recovery period was allowed between each test in the trial, and a 10-minute non-stimulation recovery period was allowed before the entire process was repeated for control trial number 2 and 3. The procedure was repeated 3 times and averaged only when all neurons met the inclusion criteria of 10% variation in action potential firing for all mechanically evoked neuronal responses.

In vivo spinal pharmacology with electrophysiological monitoring

After collection of predrug baseline control data as outlined above, atipamezole (a α_2-AR antagonist: 100 µg; Sigma-Aldrich, Gillingham, United Kingdom, dissolved in 97% normal saline, 2% Cremophor [Sigma, UK], 1% dimethyl sulfoxide [DMSO; Sigma, UK] vehicle), prazosin hydrochloride (α_1-AR antagonist: 20 µg, Sigma-Aldrich, Gillingham, United Kingdom, dissolved in water for injections) was administered topically to the spinal cord in 50 µl volumes following gentle removal of residing saline in the agarose well. Each individual drug dose effect (one stable neuron assessed per rat) was followed for up to 40 minutes with tests performed typically at 3 time points (starting at 10, 20 and 30 minutes). For each time point, a trial consisted of consecutive stable responses to von Frey and DNIC (von Frey with concurrent ipsilateral ear pinch).

Optogenetics

Light stimulation during spinal WDR recordings

The 450 nm laser (Doric Lenses, Quebec, Canada) or 637 nm laser (Thorlabs, UK) was externally TTL-triggered by the neurolog system (NeuroLog system, Digitimer, UK) to deliver defined light pulses (238 mW/mm^2 [7.5 mW], 20 ms pulse width at 5 Hz for 450 nm laser pulses to activate ChR2) or continuous illumination (at 160 mW/mm^2 [5 mW] for 637 nm laser for Jaws, or at 400 mW/mm^2 [12.5 mW] for 450 nm laser for GtACR2). As described earlier[9], the laser was coupled via a multimode 200 µm patch cord (0.39 NA, #M75L01, Thorlabs, UK) to multimode stainless steel 20 mm long cannula (200 µm diameter, 0.39 NA, #CFM12L20, Thorlabs, UK). The light power density was adjusted for each preparation (measured at the tip of implantable 200 µm cannula using power meter (PM16-130, Thorlabs, UK)). After desired power was achieved the fibre was slowly inserted

100 µm above the studied brainstem nucleus ipsilateral to the injected vectors and the recorded spinal WDR neurons. The fibre was lowered using precise hydraulic micromanipulator (Narishige, Japan) mounted on stereotaxic frame adjusted to desired coordinates to target studied nucleus (same coordinates were used as in the brainstem virus injections). For GtACR2 preparations, a second optic fibre connected to its own patch cord was positioned over the ipsilateral dorsolateral funiculus, 200-300 µm rostrally from the recording electrode. For the DLF modulation, brain ending patch cord was disconnected from the laser and replaced by the spinally terminating fibre. Light intensities were individually adjusted for both brain and spinal fibres. Optic fibres used to target nuclei were paint-coated except the tip to ensure minimum light off target effects around the fibre. Spinal WDR neurons were characterised by three stable baseline responses followed by three optically modulated responses. For combined optogenetics and spinal pharmacology, after collecting three stable baseline and three stable optoactivation responses (averaged, if stable), a drug (100 µg atipamezole) was applied topically on the exposed spinal cord surface, right above the recording site. To assess simultaneous action of the drug and the A5 optoactivation, light pulses were delivered 30 s before and throughout each series of tests (approximately 5 minutes per series) and minimally 5 minutes of the recovery time was allowed between the tests. Pharmacology was monitored every 10 minutes for 30-40 minutes (each test with optoactivation). At the end of every experiment, animals were sacrificed by the overdose of isoflurane and transcardially perfused with cold saline followed by 4% paraformaldehyde for anatomical evaluation.

A6 neuron recording and optoinhibition

A simultaneous recording and optical stimulation of the transduced A6 neurons were made using microoptrodes as described earlier[9] with minor modifications. For the A6 recordings and optoactivation, the all-glass recording microptrode with 20 µm tip diameter consisted of the recording core filled with 3 M sodium acetate (resulting in 2-3 MΩ resistance) and the parallel gradient index (GRIN) optical core (a gift from Professor Yves De Koninck, Laval University, Canada) coupled to the optic fibre (multimodal, 200 µm core diameter, 0.39 NA, #M77L01, Thorlabs, UK) was used. The GtACR2-expressing A6 neurons were optoinhibited by 450 nm laser (Doric Lenses, Quebec, Canada) light continuous illumination as described above.

Immunohistochemistry

Histochemical analysis was performed exactly as described earlier[9]. Briefly, collected brains and spinal cords were cryo-sectioned (Bright Instruments, UK) to 25 μm thick coronal slices subsequently collected on eight slides (a slice collected every 200 μm). Next, slides with odd numbers were immunostained for quantification with primary antibodies against dopamine-β-hydroxylase (DBH, a marker of noradrenergic neurons: Mouse, 1:500, Millipore, MAB308, UK), mCherry (Rabbit, 1:500, Abcam, ab167453, UK), fRed (rabbit anti-tRFP, 1:500; AB233, Evrogen), or eGFP (chicken, 1:1000, ab13970; Abcam, United Kingdom) followed by incubation with the appropriate fluorophore-conjugated secondary antibodies (Donkey anti-Rabbit, Alexa Fluor 568, A10042, Invitrogen, Eugene, OR, US; Donkey anti-Mouse, AlexaFluor 488, A21202, Invitrogen, Eugene, OR, US; all used at 1:1000 dilution). Slides were protected with mounting media (Fluoromount-G with DAPI, eBioscience, UK) and coverslips and stored in darkness at 4°C until imaging. Samples were typically imaged with an LSM 710 laser-scanning confocal microscope (Zeiss) using Zeiss Plan Achromat 10x (0.3 NA) and 20 x (0.8 NA) dry objectives and analysed with Fiji Win 64. For quantification, samples were imaged with 20x dry objective on Zeiss Imager Z1 microscope coupled with AxioCam MRm CCD camera. The acquisition of images was made in multidimensional mode and the MosaiX function was used to construct the full view. 6-8 slices on odd slides were imaged per animal. Cell counting was carried out on the Fiji Win 64 utilising cell counter plugin. On average, 20-30 brainstem sections were imaged for quantification.

Passive Tissue Clearing (PACT)

A passive CLARITY tissue clearing technique (PACT)[12] has been implemented to allow thick >1000 μm tissue fragments imaging in CAV/PRS-GtACR2-fRed injected rats. The procedure was performed as described earlier[9] with minor modifications. Briefly, after fixation samples were pre-cut using vibratome (Leica, Germany) at 600 μm coronal brainstem sections, or 800 μm coronal or sagittal spinal cord sections. Slices were then transferred directly to ice-cold A4P0 solution and treated as described earlier[9]. Following for passive clearing, samples were incubated with 500-1000 μl of rabbit anti fRed (1:500; AB233, Evrogen) primary antibody per slice in a 2 ml Eppendorf tube at room temperature, with gentle shaking for 2 days, followed by additional 2 days with the goat anti-rabbit fluorophore-conjugated secondary antibody (1:500, Alexa Fluor 647, A21244, Invitrogen, Eugene, OR, US). Finally, samples were equilibrated to the Histodenz-based (#D2158, Sigma-Aldrich, UK) refractive index-matching solution (RIMS, refractive index = 1.47) and placed in fresh RIMS in custom-made glass slide chambers for imaging. Zeiss LSM 780

confocal upright microscope, equipped with EC Plan-Neofluar 10x 0.3 NA, Ph1 dry objective (WD=5.3 mm, cat. 420341-9911, Zeiss, Germany) and 633 nm laser lines was used for imaging. Scans were taken with 2048x2048 pixel resolution, with 4-5 μm optical section typically spanning 400-700 μm of scanned depth (resulting in 100-150 planes) with auto Z-brightness correction to ensure uniform signal intensity throughout the sample. Images were exported from Zen 2012 Blue Edition software (Carl Zeiss Microscopy GmbH, Germany). Next graphical representations, 3D-rendering, animations, maximal intensity projections within selected z-stacks and further analysis were obtained with open-source Fiji (ImageJ) equipped with appropriate plugins.

Quantification and Statistical Analysis

Typically, up to 3 WDR neurons were characterised per preparation (n), and data were collected from at least 6 rats per group (N). Single pharmacological investigation was performed on one neuron per animal. Statistical analysis was performed either on number of neurons (n) for populational studies, or number of animals (N) for pharmacological studies. Detailed description of the number of samples analysed and their meanings, together with values obtained from statistical tests, can be found in each figure legend. Data represent mean ± SEM and is plotted as such (standard plots) or as median with quartiles (violin plots). Symbols denoting statistically significant differences were explained in each figure legend. Main effects from analysis of variance (ANOVA) are expressed as an F-statistic and *p*-value within brackets. Throughout, a p-value below 0.05 was considered significant. Uncorrected two-way repeated-measures (RM) ANOVA with the Tukey *post-hoc* was used to assess von Frey and DNIC responses in the baseline conditions. For pharmacological experiments, Geisser-Greenhouse correction was used for RM-ANOVA. GraphPad Prism was used to analyse the data.

Additional references:

1. Zimmermann M. Ethical guidelines for investigations of experimental pain in conscious animals. *Pain*. 1983;16(2):109-110. doi:10.1016/0304-3959(83)90201-4

2. Kilkenny C, Browne WJ, Cuthill IC, Emerson M, Altman DG. Improving bioscience research reporting: the ARRIVE guidelines for reporting animal research. *PLoS Biol*. 2010;8(6):e1000412. doi:10.1371/journal.pbio.1000412

3. Tervo DGR, Hwang B yeol, Viswanathan S, et al. A Designer AAV Variant Permits Efficient Retrograde Access to Projection Neurons. *Neuron*. 2016;92(2):372-382.

doi:10.1016/j.neuron.2016.09.021

4. Chuong AS, Miri ML, Busskamp V, et al. Noninvasive optical inhibition with a red-shifted microbial rhodopsin. *Nat Neurosci*. 2014;17(8):1123-1129. doi:10.1038/nn.3752

5. Li Y, Hickey L, Perrins R, et al. Retrograde optogenetic characterization of the pontospinal module of the locus coeruleus with a canine adenoviral vector. *Brain Res*. 2016;1641:274-290. doi:10.1016/j.brainres.2016.02.023

6. Hickey L, Li Y, Fyson SJ, et al. Optoactivation of Locus Ceruleus Neurons Evokes Bidirectional Changes in Thermal Nociception in Rats. *J Neurosci*. 2014;34(12):4148-4160. doi:10.1523/JNEUROSCI.4835-13.2014

7. Mahn M, Gibor L, Patil P, et al. High-efficiency optogenetic silencing with soma-targeted anion-conducting channelrhodopsins. *Nat Commun*. 2018;9(1). doi:10.1038/s41467-018-06511-8

8. Hayat H, Regev N, Matosevich N, et al. Locus coeruleus norepinephrine activity mediates sensory-evoked awakenings from sleep. *Sci Adv*. 2020;6(15). doi:10.1126/sciadv.aaz4232

9. Kucharczyk MW, Di Domenico F, Bannister K. Distinct brainstem to spinal cord noradrenergic pathways inversely regulate spinal neuronal activity. *Brain*. 2022;(March):1-18. doi:10.1093/brain/awac085

10. Bannister K, Patel R, Goncalves L, Townson L, Dickenson AH. Diffuse noxious inhibitory controls and nerve injury: restoring an imbalance between descending monoamine inhibitions and facilitations. *Pain*. 2015;156(9):1803-1811. doi:10.1097/j.pain.0000000000000240

11. Kucharczyk MW, Derrien D, Dickenson AH, Bannister K. The Stage-Specific Plasticity of Descending Modulatory Controls in a Rodent Model of Cancer-Induced Bone Pain. *Cancers (Basel)*. 2020;12(11):1-17. doi:10.3390/cancers12113286

12. Treweek JB, Chan KY, Flytzanis NC, et al. Whole-body tissue stabilization and selective extractions via tissue-hydrogel hybrids for high-resolution intact circuit mapping and phenotyping. *Nat Protoc*. 2015;10(11):1860-1896. doi:10.1038/nprot.2015.122
